# Letters from Paris

**Published:** 1870-02

**Authors:** A. W. Bosworth

**Affiliations:** Paris


### Foreign Correspondence.

PARIS, *December* 30, 1869.

IN a medical way every thing is now in full tide. The various learned societies are more or less occupied with discussions on the transmission of syphilis by vaccination; on the treatment of syphilis by mercury, or by hygiene, tonics and time.

A new chair, entitled, "History of Medicine," has lately been created at the medical school, after a somewhat stormy discussion among the professors.

One of the clinical professors of the faculty, on his return from his summer vacation, being informed that his house physician was having fair success in the treatment of typhoid fever by the cold douche, said that he knew something far more efficacious. It consisted in putting the typhoid patient into a cold bath, and gradually reducing the temperature to a low degree. This was done to three unfortunate individuals, who very soon succumbed, and the professor quietly renounced his unsuccessful experiment.

As there is nothing of great interest to write concerning hospital service, we will give a part of a report presented at the Academy of Sciences, on the 6th of this month, by M. Andral, concerning the variations of temperature of the human body, with variations of some of the principal constituents of the blood.

The temperature was taken in the axilla. He said that when the blood contains more than four thousandths of fibrin the temperature rises. That the temperature and fibrin augment in direct proportion to each other. Thus, of all diseases which increase the quantity of fibrin, pneumonia causes the greatest excess, and it is this disease also which produces the most heat. In 85 cases of pneumonia, 13 times he found the temperature below 39° centigrade;* 44 times from 39° to 40°; 21 times 40°; once 41°; twice 41°2′.

In acute pleurisy he has always found the quantity of fibrin less than in pneumonia, and has seen the temperature only exceptionally surpass 40°, presenting for a maximum, in one case only, 41°. Most ordinarily it vacillated between 39°5′ and 38°5′.

In acute capillary bronchitis the fibrin is generally less than in pleurisy, and the temperature he has never seen exceed 39°.

In acute articular rheumatism, which, next to pneumonia, augments the most the quality of fibrin, he has never seen the temperature reach 41°. In this disease the maximum seen was 40°5′; generally it is between 39° and 40°.

Finally, although in some cases of acute pulmonary phthisis the temperature may reach 40°5′, it remains, in ordinary cases that have reached the stage of continued fever, between 38° and 39°5′, which is in rapport with the fibrin; the latter maintaining itself between four and five thousandths. This general fact has, nevertheless, its exceptions. Thus, in erysipelas, where the highest quantity of fibrin found was $\frac{7}{1000}$, he has seen the temperature rise to 41°8′. In other cases of the same disease it was 41°2′,

* All the degrees here used are centigrade. If the editor chooses, can give the rule for reducing centigrade to Fahrenheit.

40°6′, 40° and 39°. Other phlegmasias have presented similar exceptions — thus, 40° with only $\frac{4}{1000}$ of fibrin. There are, nevertheless, very large quantities of fibrin which he has only found with certain very high degrees of temperature. Such is the number 10, which he has only seen appear when the temperature had surpassed 40°. But, one may ask, what kind of rapport exists between this simultaneous increase of heat and fibrin? Is it a simple coincidence? Is it casualty? To this question the answer is easy, on account of there being one large class of diseases — pyrexias — in which the fibrin rests in its physiological limit, can even descend below it, and where the temperature is as considerable, and perhaps more, than in the phlegmasias which characterize an augmentation of fibrin. It is, in fact, in these pyrexias that he has found the maximum of temperature, viz.: 3 times 42°; once 42°4′. He found this last degree in a case of typhoid fever; that of 42° in the primary fever of variola, in the period of heat of an access of intermittent fever, and in a case of glanders in man.

He says that the fibrin does not augment in eruptive fevers; and, nevertheless, he has found, in the invading fever of small-pox, as a minimum of temperature, 40°; after, the numbers 40°5′, 40°9′, 41°, 42°; that is to say, numbers equal or superior to those observed in diseases where there is the greatest increase of fibrin.

In the invading fever of scarlatina he has found the temperature oscillating between 39° and 40°7′. It was less in measles, maintaining itself in the invading fever between 39° and 37°7′, and during the eruption between 38° and 40°5′.

From these results, he says, we may conclude that the increase of fibrin and of temperature are only two facts, which, in certain diseases, are produced together, without one depending on the other; that there is so little between these two facts, a rapport of casuality, that the augmentation of temperature is raised to its highest degree, in the morbid states of which one of the characters is a tendency to a diminishing of the plastic element of the blood.

He next examined the question, Does the quantity of globules exercise any influence on the temperature? The facts which are going to be exposed show that a diminution, even very great, of the number of globules, does not cause the temperature to descend below the inferior limit of a physiological state. Sometimes, then, we see it approach this inferior limit; at other times rise towards the superior limit, and even surpass it a little.

A woman reduced by frequent and abundant hæmorrhage, caused by uterine cancer, had no more than 21 parts of globules in her blood; nevertheless, her temperature continued at 37°.

A man, very anæmic from long mercurial treatment, had only 87 globules in his blood. His temperature was 36°7′.

In a case of saturnine cachexia, where the number of globules were not more than 83, the temperature had risen to 38°.

A patient, affected with scurvy, had only 44 globules in his blood; nevertheless, he had a temperature of not less than 38°.

In chlorosis the same fact is seen; and, whatever may be the diminution of globules, the temperature does not descend below the physiological state, but, sometimes, even rises above it.

A chlorotic woman, who had 38 grammes of globules for 1,000 grammes of blood, had, notwithstanding, 37°9′.

The following table, proving the above assertions, shows number of globules and the temperature taken from twenty chlorotic persons:

| GLOB. | TEMP. | GLOB. | TEMP. | GLOB. | TEMP. |
|---|---|---|---|---|---|
| 38 | 37°9′ | 62 | 37°9′ | 104 | 37°0′ |
| 46 | 37°9′ | 64 | 37°0′ | 104 | 38°0′ |
| 48 | 38°4′ | 77 | 37°5′ | 112 | 37°7′ |
| 49 | 38°0′ | 86 | 37°5′ | 112 | 37°5′ |
| 49 | 37°6′ | 95 | 37°3′ | 113 | 38°0′ |
| 54 | 37°7′ | 97 | 38°1′ | 117 | 37°7′ |
| 56 | 38°0′ | 99 | 37°9′ | | |

With these 20 cases the minimum degree was 37, and this only twice; with three it was 37°5′; with the 15 others it was between 37°6′ and 38°4′, without, in any case, the existence of an intercurring inflammatory lesion explaining these elevated numbers.

These facts prove, in a physiological point of view, that the red globules of the blood can vary much in quantity without the animal heat being modified; that, in a pathological point of view, these maximums of normal temperature, and even these commencements of morbid temperature with a certain number of chlorotic persons, enable us to comprehend those sensations of troublesome heat, as if febrile, which many of them *eprouve*, and justifies, to a certain degree, the expression of "Fever of Chlorotic Persons," employed by some nosographs.

In my next shall expose his views in regard to albumen and urea. A. W. Bosworth.

---

Paris, *January* 13, 1870.

*Albumen.*—In my last, I promised you the rest of M. Andral's report to the Academy. He said that when the albumen of the blood, instead of being completely employed for nutrition, is in part lost for it by its being passed off in the urine, theory would seem to indicate that less heat should be produced; and some facts here mentioned would authorize, if they were more numerous, that such were really the case.

In seven cases of albuminuria, where he noted the temperature, there were two having temperature notably below the normal state, being from 35° to 35° 3′; in thirty other cases, the temperature was from 36° to 36° 5′; in two cases there was elevation of temperature, being 38° to 39°. Of these two latter cases, in the first the albuminuria was complicated by an acute inflammation of the lymphatic glands of the neck, which terminated by suppuration. In the second, the temperature was taken at the beginning of the disease, which, contrary to the general rule, had signalized its apparition by symptoms of acute nephritis, with complication of erysipelas of the face; consequently, these two cases were only in apparent discord with the fifty others, their elevation of temperature having a sufficient cause.

The pathology of facts which he mentions prove that it is not immediately, but, on the contrary, after some time, that the insufficient albuminous material decreases the temperature in an appreciable manner. Thus, with convalescents who have just undergone dieting for a few days, we do not find the temperature so much diminished as might be expected. The lowest degree found in such cases was 36°7′; it was usually 37°, or between 37° and 37°5′.

The author has often been surprised to find that heat remained physiologically normal with patients affected with cancer of the stomach, who daily vomited the greater part of their food. Nevertheless, a moment arrives when the temperature, after having long been maintained at a normal degree, in spite of almost complete absence of reparatory substances, diminishes suddenly. He has seen it thus fall in twenty-four hours from 37° to 35°.

These facts are in accordance with the experiments of Chossart. The animals, which he caused to die of hunger, maintained their normal temperature till two or three days before death, when it suddenly diminished.

*Urea.*—To decide whether urea contained in the urine augments during febrile diseases, is a much-discussed question. It is difficult to decide if we attempt to take for a starting-point the normal quantity of urea said to be in the urine, because that is yet only known in a very uncertain manner. Thirty grammes of urea for every 1,000 grammes of urine, given formerly by Berzelius, is evidently too high. Andral thinks from 10 to 14 and 15 grammes is the most that can be admitted. He believes to obtain more accurate results by examining comparatively the quantity of urea of patients having a normal temperature with those having it augmented. In fifty-three of the former cases, he only found eight times more than 8 grammes of urea to 1,000 grammes of urine, viz.: 14, 17, 22 and 23 grammes, in four cases of organic disease of the heart; 19 grammes, in one case of cancer of the stomach; 20 to 22 grammes, in three cases of cirrhosis of the liver. In the forty-five other cases, which only had absence of fever as the only similarity. the greatest quantity of urea was 12 grammes; the minimum, 4 grammes. In these fifty-three cases, the urea often varied in a notable manner, in the same patient, at short intervals. Ex.: In a case of disease of the heart, it was, within a few days, from 4 and 8 to 22 grammes. If we now compare the preceding cases with those having fever, we find with these latter a greater quantity of urea, a greater constancy of this quantity, and, in general, a proportional rapport between the quantity of urea and the degree of temperature. In these affections, all pyretical, he found, as a maximum of urea, 40 grammes — this in a case of urticaria, with much fever.

One of the morbid states in which the temperature is seen to rise most, is intermittent fever. In twenty-three cases of this fever, he found the urine to contain eleven times from 20 to 32 grammes of urea; nine times, from 16 to 20 grammes; twice, only, to fall below this last number, being once 11 grammes and once 13.

In pneumonia, the urea vacillated between 20 and 29 grammes, with the exception of one case, where it was only 9 grammes. In this latter case, the pneumonia affected only a small number of lobules, and there was very little fever.

Pleurisy, which augments less the temperature than pneumonia, gives, also, less urea. In pleurisy, its maximum was 18 grammes; then came the numbers 14 and 15.

In acute articular rheumatism, he found, when the fever augmented, there was, at the same time, an increase of urea, of which was found 18, 19, 20, 27, and 31 grammes. When the fever decreased, there was then only 16, 15, 14 grammes of urea. When it had completely ceased, the urea descended from 14 to 8 grammes; finally, after six weeks or two months, the patients, weakened by prolonged dieting, by extraction of blood, by pain, returned slowly to health, the urea, still diminishing, fell sometimes to 6, 5, 4, and even 3 grammes.

He had only three cases of *typhoid fever* to present. Here, also, the urea augmented with the temperature, and decreased with it. In the first case, when the temperature was 40°, there was found 28 grammes of urea in the urine. In the two other cases, when the temperature was taken in the decline of the disease, and was 38°5′, there was only 13 and 12 grammes of urea. From these cases, he is unwilling to accept the opinion of some authors, who state that, in typhoid fever, the urea diminishes instead of increasing.

We must not forget that diet acts in an opposite manner to fever; therefore it may happen that, when the fever has lasted a long time, the urea, without ceasing to be considerable, becomes less, the elevation of temperature resting the same.

He possessed only one analysis of the urine for eruptive fevers, which was confirmative of what has been here asserted. The urine contained 30 grammes of urea on the second day of the invading fever of variola.

The fever of phthisical patients offers a temperature generally inferior to that of the acute inflammations of fevers, and also gives a less quantity of urea. The greatest quantity found in phthisis was 14 grammes; the most frequently, 8 to 12 grammes; and, with some patients affected by this disease, when, having arrived at the last degree of marasmus, the urea had decreased, in spite of the fever, to 6, and even 4 grammes.

The radical weakening of the constitution, the prolonged, insufficient alimentation, the daily losses of expectoration, perspiration and diarrhœa, can explain how alone of all the febrile diseases, pulmonary tuberculation can raise the temperature, without always augmenting the urea.

It is more difficult to explain those inverse cases where, the temperature resting normal, the urea accidentally rises to those quantities found in a febrile state. The most diverse affections can present this anomaly, which must depend on some individual disposition of the patients.

There is, nevertheless, one morbid state, cirrhosis of the liver, in which, in the cases where he examined the urine, he found the urea increased. If other observations should confirm these results, we must conclude that, contrary to the other apyretic maladies, cyrrhosis of the liver increases the secretion of urea, not accidentally, but by its nature. If this exception exists, what may be its cause? Can we suppose that the azotized material of the bile, which, no longer being able to leave the blood through the altered tissue of the liver, finds a supplementary exit of elimination in the kidneys? This would be the inverse of what occurs when M. Claude Bernard, suppressing the renal secretion, finds an unusual quantity of highly azotized matter in the interior of the digestive canal.

M. Bouillaud then addressed the Academy, remarking that the results of his long researches perfectly coincide with what M. Andral had said.

Mr. Editor: In my next I may possibly give you some notes from Brown-Sequard. He is now lecturing on epilepsy. His course is not so well attended as might have been expected.

With kind regards, I remain your humble servant,

A. W. Bosworth.

---

The womb, according to Plato, " is a wild beast that obeys no reason, but which, when its desires are unsated, wanders about within the body, and excites all sorts of irregular motions." " Our ancient brethren," says Dr. Taliaférro, " believed the uterus to possess an independent existence, and the body to be but a cage for its confinement."

